# Structural mechanisms of Tad pilus assembly and its interaction with an RNA virus

**DOI:** 10.1126/sciadv.adl4450

**Published:** 2024-05-03

**Authors:** Yuhang Wang, Matthew Theodore, Zhongliang Xing, Utkarsh Narsaria, Zihao Yu, Lanying Zeng, Junjie Zhang

**Affiliations:** Center for Phage Technology, Department of Biochemistry and Biophysics, Texas A&M University, College Station, TX 77843, USA.

## Abstract

*Caulobacter crescentus* Tad (tight adherence) pili, part of the type IV pili family, are crucial for mechanosensing, surface adherence, bacteriophage (phage) adsorption, and cell-cycle regulation. Unlike other type IV pilins, Tad pilins lack the typical globular β sheet domain responsible for pilus assembly and phage binding. The mechanisms of Tad pilus assembly and its interaction with phage ΦCb5 have been elusive. Using cryo–electron microscopy, we unveiled the Tad pilus assembly mechanism, featuring a unique network of hydrogen bonds at its core. We then identified the Tad pilus binding to the ΦCb5 maturation protein (Mat) through its β region. Notably, the amino terminus of ΦCb5 Mat is exposed outside the capsid and phage/pilus interface, enabling the attachment of fluorescent and affinity tags. These engineered ΦCb5 virions can be efficiently assembled and purified in *Escherichia coli*, maintaining infectivity against *C. crescentus*, which presents promising applications, including RNA delivery and phage display.

## Teaser

Structures of Tad pili and the ΦCb5 phage–pilus complex elucidate mechanisms for pilus assembly and phage adsorption.

## INTRODUCTION

Bacterial pili, hair-like appendages present on the surfaces of various bacteria, perform critical roles including adhesion, colonization, motility, and conjugation/competence, and can act as receptors for bacteriophage (phage) binding (*1*–*3*). Understanding the functions and diversity of bacterial pili is essential in studying bacterial pathogenesis and developing strategies to combat bacterial infections. *Caulobacter crescentus*, a Gram-negative aquatic bacterium, is known for its distinctive cell cycle, characterized by asymmetric division and two distinct morphological forms: the swarmer cell and the stalked cell (*4*, *5*). It has the tight adherence (Tad) pili, which are primarily produced in the swarmer cells. Tad pili of *C. crescentus*, also known as type IVc pili, belong to the type IV pili family and are involved in surface sensing/adherence (*6*, *7*) and biofilm formation (*8*). Driven by a bifunctional adenosine triphosphatase, Tad pili exhibit a dynamic process of extension and retraction (*9*). Upon surface sensing, pili retraction reorients cells into an upright position, promoting walking-like movements (*10*). This aids in positioning the flagellate pole close to the surface, anchoring the holdfast, a durable adhesive structure to ensure long-term attachment. The retraction of Tad pili leads to disassembly of pilin subunits, which are subsequently recycled to the pilin reservoir in the inner membrane. These recycled pilins are proposed to interact with PleC kinase to increase cyclic diguanylate monophosphate production and initiate holdfast synthesis, thereby linking Tad pili dynamics with cell-cycle regulation (*7*, *11*). Notably, the holdfast synthesis can also be stimulated by resistance of pili retraction, without surface contact (*6*). Unlike other classes of type IV pili, the mature Tad pilin sequences are notably shorter, aligning solely with the N terminus of other mature type IVa (*12*) and archaeal type IV pilins (fig. S1, A and B) (*13*). The assembly and disassembly of such “truncated” pilin subunits, which promote surface adaptation of *C. crescentus*, remain a mystery. The structure of Tad pili is crucial to explain the mechanism that underlies their diverse functions and significance in cell physiology.

Furthermore, *C. crescentus* Tad pili are proposed as a phage receptor and indispensable for the infection of a single-stranded RNA (ssRNA) phage, ΦCb5 (*14*, *15*). ΦCb5 carries a 3762-nt RNA genome encoding essential proteins, including a maturation protein (Mat), a coat protein (Coat), the β subunit of an RNA-dependent RNA replicase (Rep), and a lysis protein (Lys), with the gene *lys* embedded in the reading frame of the *rep* gene (*16*). It is proposed that these ssRNA phages attach to the side of retractile pili using Mat and enter bacteria during pili retraction (*15*). However, in contrast to other type IV pilins (*12*, *13*, *17*), Tad pilins lack a surface-exposed globular β sheet domain used by other ssRNA phages, such as PP7, for binding (*18*). This distinction suggests that the adsorption mechanism between ΦCb5 and Tad pili may deviate from other phage-pilus pairs. Such assumption remains untested, primarily because of the lack of a structure of the mature ΦCb5 virion as well as a structure of the ΦCb5-Tad complex.

In this study, we use single-particle cryo–electron microscopy (cryo-EM) to determine the molecular structure of the *C. crescentus* Tad pilus and comprehensively examine the molecular basis for the interaction between Tad pili and ΦCb5. These findings not only present the structure of Tad pilus but also offer invaluable insights into the Tad pilus assembly and adsorption mechanisms of ssRNA phages in general. Furthermore, these data have inspired the design of a versatile phage production platform, enabling tailored customizations of these ssRNA phage particles for research and technological applications.

## RESULTS

### Molecular structure of the *C. crescentus* Tad pilus

**Fig. 1.**
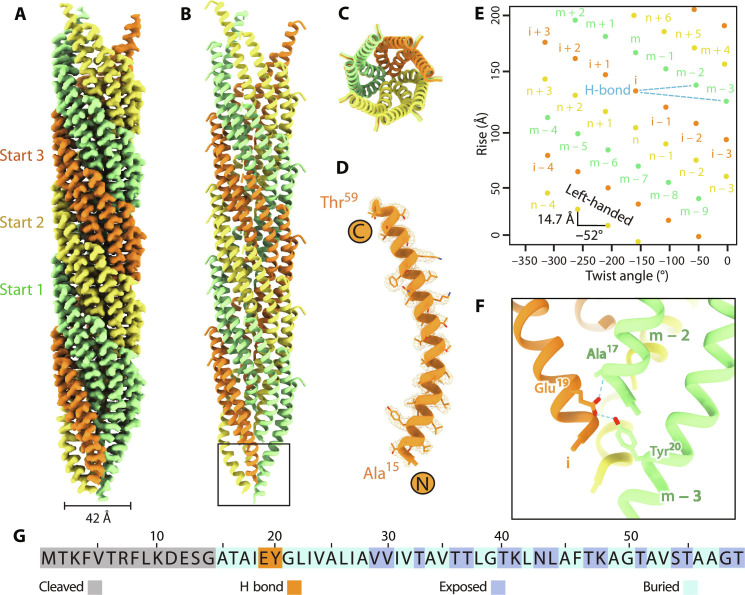
Cryo-EM structure of the *C. crescentus* Tad pilus. (**A**) Cryo-EM density map of the Tad pilus with each strand of the three-start helix colored as green, yellow, and orange, respectively. (**B**) Atomic model of the Tad pilus. (**C**) The top end-on view of the Tad pilus showing interactions between adjacent pilins. (**D**) Atomic model of a single pilin fits in the density map at a contour level of 0.394σ. The N- and C-terminal residues are labeled. (**E**) The assembly pattern plot showing the helical rise (14.7 Å) and twist angle (−52°) in the Tad pilus. Each dot represents a pilin and with the color as defined in (A). Each strand of the three-start helix is named m, n, and i, respectively, and numbered in a left-handed fashion. Each pilin forms two hydrogen bonds (blue dashed lines) with two pilins in the neighboring strand on the right at the −2 and −3 positions, respectively. (**F**) Close-up view from the boxed region in (B) showing two hydrogen bonds (blue dashed lines) from the Glu^19^ on Pilin (i) to the backbone of Ala^17^ on Pilin (m − 2) and the Tyr^20^ on Pilin (m − 3), respectively. (**G**) The sequence of *C. crescentus* bNY30a strain Tad pilin shows the position of cleaved residues, exposed residues, and buried residues.

To unveil the structural and assembly mechanisms of Tad pili, we purified Tad pili from the hyperpiliated bNY30a strain of *C. crescentus* host and determined their structure at a resolution of 2.8 Å (fig. S2). The Tad pilins self-assemble into a left-handed three-start helix with a width of 42 Å, a helical rise of 14.7 Å, and a twist angle of −52° (Fig. 1).

The signal peptide comprises the first 14 amino acids of the Tad prepilin and undergoes cleavage (*19*, *20*), resulting in 45 residues (Ala^15^ to Thr^59^) that form the mature Tad pilin (Fig. 1G). This mature pilin adopts an amphipathic α-helical conformation with the N-terminal end of the α helix pointing toward the center of the Tad pilus, while the C-terminal end extends outward.

We label each strand of the three-start helix as m, n, and i (from lower left to top right) and number the pilins within each strand in a left-handed sequence (Fig. 1E). Each pilin forms hydrophobic interactions with six neighboring pilins (fig. S3, A to H), primarily through surface-buried residues (Fig. 1G). Notably, the residue Glu^19^ at the N terminus of each mature pilin is involved in the hydrogen bonding with residues in two pilins in the right neighboring strand. For example, Glu^19^ of Pilin (i) forms one hydrogen bond with the backbone of Ala^17^ of Pilin (m − 2) and another hydrogen bond with Tyr^20^ of pilin (m − 3) (Fig. 1, E and F). Owing to the helical symmetry inherent in the Tad pilus, these hydrogen bonds create an intricate network of interactions at the core of the entire pilus, precisely at the N terminus of neighboring pilins (fig. S3, J and K), which bolster the overall stability of the pilus assembly. Notably, sequences of Tad pilins exhibit remarkable conservation across various Tad pili species, particularly for these two residues, Glu^19^ and Tyr^20^ (fig. S1C), involved in the hydrogen bonding. In total, Glu^19^ and Tyr^20^ are crucial for the assembly of Tad pili, but not pilin expression or processing (*19*), which strongly suggest the requirement of these two hydrogen bond forming residues in the proper assembly of Tad pili.

### Cryo-EM structures of the ΦCb5 virion

To elucidate the structural mechanism governing the interaction between ΦCb5 and Tad pili, we conducted single-particle cryo-EM of purified ΦCb5 mixed with purified Tad pili. This enabled us to determine two distinct structures: one representing the apo ΦCb5 virion and the other showcasing the ΦCb5 virion adsorbed to a Tad pilus (fig. S4).

Our current structural understanding of ΦCb5 is limited to its Coat (*21*). The complete structure of the virion, including the 3762-nt guide RNA (gRNA) and the Mat, remains undiscovered. With our cryo-EM map of the ΦCb5 at a resolution of 2.7 Å, we reveal insights into the RNA packaging within the mature ΦCb5 virion and the arrangement of the Mat responsible for interacting with Tad pili.

**Fig. 2.**
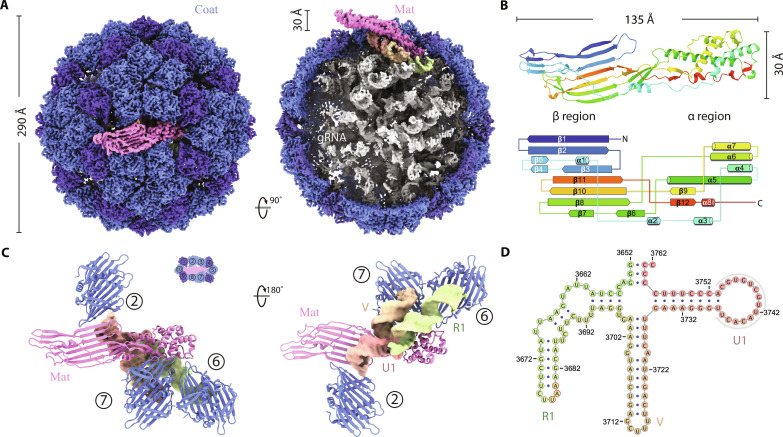
Structure of the ssRNA phage ΦCb5 mature virion. (**A**) A composite cryo-EM density map of ΦCb5, assembled with the virion and an additional focused-refinement map around the Mat (pink), in the top view (left) and the cut-open side view (right). The gRNA density is overall gray except for the last 111 nucleotides (colored differently, including the last stem-loop of the replicase RNA gene and the 3′ untranslated region, or 3′UTR), which forms a four-way junction domain and interacts with the Mat. (**B**) The secondary structure model (top) and diagram (bottom) of the Mat with all the α helices and β strands labeled. (**C**) The 3′ end domain of the gRNA (colored density) interacts with the Mat (pink model) and 3 of the 10 Coat dimers (blue models) around the Mat. The numbering of the 10 Coat dimers around the Mat is defined in the inset. Two views are presented in this region from outside (left) or inside (right) the capsid. (**D**) The secondary structure of the 3′ end (residues 3652 to 3762) of the gRNA, which forms a four-way junction domain with the three long stem-loops labeled R1, V, and U1, respectively. The base pairing in the tip of U1, which could not be verified by the cryo-EM density, is omitted and outlined by a gray arch.

The ΦCb5 virion showcases a near-icosahedral spherical Coat shell of 89 Coat dimers with a diameter of around 290 Å, while a single Mat extends 30 Å beyond the shell (Fig. 2A). The gRNA adopts a defined conformation, presenting stem-loops to interact with the inner surface of the capsid. In comparison to the Coat shell of other ssRNA phages, such as the coliphages MS2 and Qβ, ΦCb5 presents a larger opening at the pseudo sixfold axes (fig. S5A). In addition, our structural analysis reveals the presence of density corresponding to calcium ions throughout the mature ΦCb5 virion (fig. S5, B and C). This observation aligns with previous research indicating that calcium ion plays a crucial role in stabilizing the ΦCb5 Coat shell (*21*).

The Mat of ΦCb5 measures 135 Å long and 30 Å wide. It comprises a structural framework featuring a large β sheet including β strands β1 to β8, β10, and β11 and designated as the β region, as well as an α helix bundle, encompassing α helices α3 to α8 and designated as the α region (Fig. 2B). The α region and β region are connected by α1, α2, β9, β12, and flexible loops. Positioned at a twofold axis of the capsid, the β region protrudes outward, while the α region penetrates deep into the capsid to bind the gRNA. In addition, we find that the N terminus of the Mat is exposed externally on the capsid, affording flexibility for potential protein tagging and engineering at this site.

The 3′ end of the gRNA forms a domain containing RNA stem-loops emerging from a four-way junction (Fig. 2D and fig. S6A). After numbering the Coat dimers around the Mat clockwise from 1 to 10, we observe that stem-loops R1 and V interact with Coat dimers 6 and 7, respectively (Fig. 2C). A closer examination of the interface between these RNA stem-loops and the Coat dimers reveals conserved interactions, involving Coat residues Arg^46^, Arg^61^, Asn^63^, and Tyr^84^, which form hydrogen bonds and salt bridges with the RNA stem-loops R1 and V (fig. S6, C and D). These Coat-dimer residues likely play a role in interacting with the remainder of the gRNA as a packaging motif.

Stem-loop U1, the final RNA helix within the gRNA genome, engages in extensive interactions with the inner surface of Mat β region with its tip sandwiched between Mat and Coat dimer 2 (Fig. 2C). The region of the Mat that interacts with stem-loop U1 is highly positively charged, which facilitates its binding to the negatively charged RNA backbone (fig. S6E). The U1-Mat interaction is conserved among ssRNA phages, as observed in coliphages like MS2 (*22*) and Qβ (*23*).

### Molecular interface between ΦCb5 and Tad pili

Pilus adsorption marks the initial phase in the ssRNA phage infection cycle and plays a pivotal role in defining phage specificity toward their respective hosts. The structure of *C. crescentus* Tad pili substantially distinguishes it from other reported pilus structures, such as conjugative pili or other classes of type IV pili (*24*, *25*). This structural divergence suggests that the recognition of Tad pili by phages like ΦCb5 may be defined by distinct molecular interactions.

**Fig. 3.**
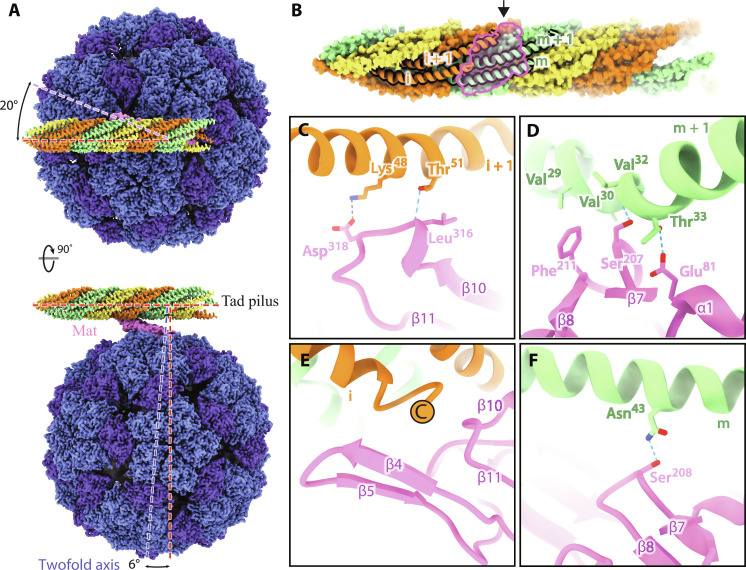
Structure of the complex between ΦCb5 and the Tad pilus. (**A**) A composite cryo-EM density map of the ΦCb5-Tad complex, assembled from the Tad pilus map, the focused-refinement Mat-pilus map, and the high-resolution ΦCb5 virion map. In the top view (top), the pilus is at an angle of 20° with the direction of the Mat (dashed pink line). In the side view (bottom), the twofold axis (dashed blue line) of the phage Coat shell is at an angle of 6° from the direction that is perpendicular to the pilus. Dashed red lines denote the directions parallel and perpendicular to the pilus. (**B**) The Mat-pilus interface area (defined by the pink outline) viewed from the Mat side. Four pilins involved in the interaction with Mat are shown as ribbon models and labeled i, i + 1, m, and m + 1, respectively. (**C** to **F**) Zoom-in views [from the direction of the black arrow in (B)] for each of the four pilins showing its interaction with the Mat (purple model). The interacting residues and secondary structures are labeled. In (E), the C terminus of the Pilin (i) is labeled by an encircled letter C.

To precisely delineate the binding interface between ΦCb5 and Tad pili, we analyze the cryo-EM structure of the ΦCb5-Tad complex (Fig. 3A). ΦCb5 attaches itself to the side of the Tad pilus, presenting a swiveling angle of 20° and a tilting angle of 6° between the pilus and the phage particle. The outer surface of the Mat β region establishes direct interactions with the exposed regions of four pilin subunits (labeled i, i + 1, m, and m + 1) from two neighboring strands of the three-start helical filament of the Tad pilus (Fig. 3B).

These four pilins engage in a complex network of interactions with the β region of Mat. In this intricate interplay, the loop linking β10 and β11 of Mat forms a hydrogen bond with Pilin (i + 1), connecting Thr^51^ of the pilin with the backbone of Leu^316^ on Mat, alongside a salt bridge involving Lys^48^ on the pilin and Asp^318^ on Mat (Fig. 3C). Moving on to Pilin (m + 1), it partakes in more extensive interactions. Specifically, it engages with the loop connecting β7 and β8 and the N-terminal end of α1 on Mat (Fig. 3D). Notably, Phe^211^ of Mat inserts into a hydrophobic pocket formed by a cluster of valines, including Val^29^, Val^30^, and Val^32^ on the pilin. In addition, Glu^81^ and Ser^207^ on Mat form hydrogen bonds with Thr^33^ and the backbone of Val^30^ on the pilin, respectively. The interaction between Pilin (i) and Mat occurs at the C terminus of the pilin, where it inserts into a pocket on Mat that is created by β4, β5, and the loop connecting β10 and β11 (Fig. 3E). Last, the loop connecting β7 and β8 of Mat interacts with Pilin (m) via a hydrogen bond between Ser^208^ of Mat and Asn^43^ of the pilin (Fig. 3F).

Upon comparing Tad pilin (PilA) sequences between the *C. crescentus* host strain bNY30a and the non-host strain NA1000, PilA_NA1000_ exhibits four mutations (fig. S7A, PDB 8U1K). Notably, two of these mutations, N43R and T51A, directly affect the hydrogen-bonding interactions with Mat. This alteration in the interacting residues of the receptor pilus could have compromised the adsorption of ΦCb5 to the Tad pilus from NA1000, contributing to its resistance to the phage. In an adsorption assay, ΦCb5 readily binds to the bNY30a strain, whereas its attachment to the NA1000 strain closely resembles that of a Tad pilin (PilA) knockout mutant strain of *C. crescentus* (fig. S7, B and C).

By inspecting our structures, Thr^36^ of all pilins on the Tad pilus are outside of the binding interface with Mat (inset of fig. S7C). This allowed cysteine mutation of Thr^36^ of PilA to fluorescently track the dynamics of Tad pili via maleimide dye labeling (*26*), without compromising their interaction with ΦCb5.

### Infectious ΦCb5 with engineered Mat assembled in *Escherichia coli*

**Fig. 4.**
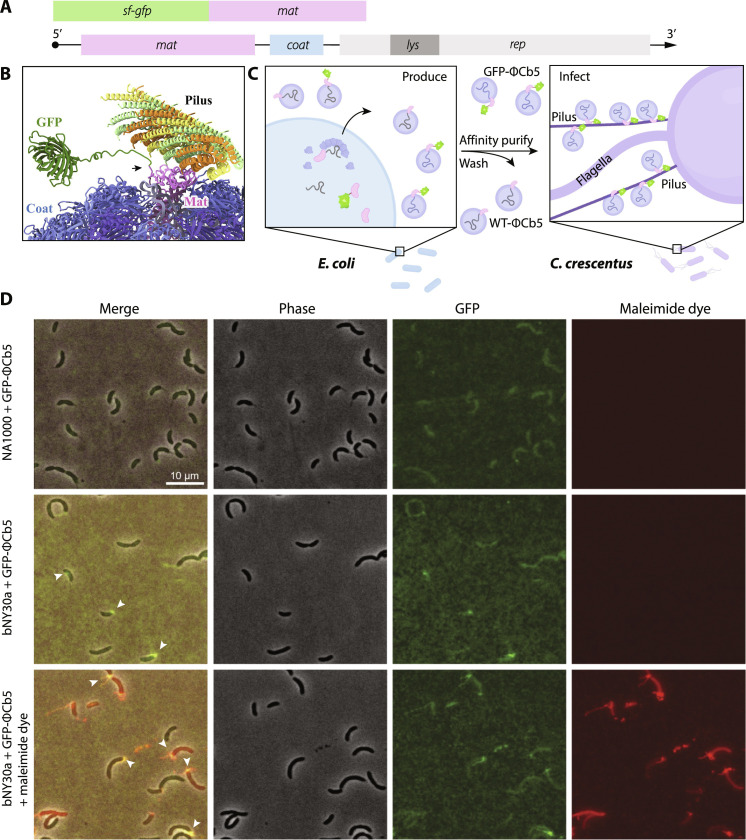
The engineered GFP-ΦCb5 can be produced in *E. coli* and bind to Tad pili of *C. crescentus* host strain bNY30a. (**A**) Schematic diagrams of the two-plasmid systems to be coexpressed in *E. coli*. Plasmid 1 contains the genes for the sfGFP and the Mat. Plasmid 2 contains the complete genome of ΦCb5 including the genes for the Mat (*mat*), Coat (*coat*), and the Replicase (*rep*). The lysis gene (*lys*) is embedded in the reading frame of *rep*. (**B**) A model showing the N terminus (indicated by a black arrow) of the Mat is exposed from the Coat shell and free from the pilus-Mat interface. This site can be engineered to fuse other proteins such as GFP. (**C**) A schematic describing the production of engineered GFP-ΦCb5 from *E. coli*, which will be affinity-purified using anti-GFP nanobodies, to infect the *C. crescentus* host strain bNY30a. The schematic was created with BioRender. (**D**) Colocalization of GFP-ΦCb5 and the Tad pili from *C. crescentus* host strain bNY30a with a T36C mutation for Alexa Fluor-594 C5-maleimide dye (red) labeling. This strain also has holdfast genes knocked out to avoid nonspecific maleimide dye labeling, which interferes with Tad pilus visualization. The non-host strain NA1000, which makes Tad pili that do not bind to ΦCb5, is used as a control. White arrows indicate the fluorescence colocalization of ΦCb5-Tad.

From our structures, the capsid-exposed N terminus of the Mat does not participate in the interaction with the Tad pilus. This opens up the possibility for engineering modifications at this site without the risk of interfering with its interaction with the pilus. We can introduce additional protein domains into ΦCb5 virions. As a proof of concept, we fused super-folder green fluorescent protein (sfGFP) to the ΦCb5 Mat. Specifically, we constructed a plasmid fusion of sfGFP with Mat, allowing for coexpression of this engineered Mat in *E. coli* alongside the ΦCb5 genome plasmid (Fig. 4A). Consequently, this results in the production of engineered ΦCb5 particles displaying sfGFP on the Mat (referred to as GFP-ΦCb5; Fig. 4B), in addition to the wild-type ΦCb5 virions. These GFP-ΦCb5 particles can be efficiently purified through affinity-based chromatography using anti-GFP nanobodies (Fig. 4C, fig. S8A, and Materials and Methods).

These GFP-ΦCb5 particles effectively bind and label the Tad pili of the live *C. crescentus* bNY30a cells (Fig. 4D and fig. S8B). These engineered GFP-ΦCb5 preserve infectivity against bNY30a (fig. S8C). This indicates both the successful packaging of wild-type gRNA within the capsids of the engineered GFP-ΦCb5 and the effective delivery of this gRNA into the host cells. The RNA transcribed from the GFP-Mat plasmid lacks the 3′ end domain of the wild-type ΦCb5 gRNA, responsible for binding to the Mat, which, in turn, escorts the RNA into the cell via pilus retraction. Consequently, even if the GFP-Mat RNA were to be enclosed within our engineered GFP-ΦCb5 particles, they would not be delivered into the host cell.

The successful assembly of engineered GFP-Mat onto ΦCb5 phages in *E. coli* offers advantages for large-scale production and rapid affinity-based purification. These benefits will greatly enhance their applicability in research and various technological applications. In this initial design, the WT-ΦCb5 RNA genome is transcribed from the DNA plasmid and encapsulated within these engineered GFP-ΦCb5 particles to maintain the integrity of the gRNA sequence, structure, and function. However, the presence of the WT-ΦCb5 RNA can lead to the production of WT-Mat, competing for cellular resources to produce the engineered Mat. In our two-plasmid system, we observed that the levels of incorporated WT-Mat and GFP-Mat are of a similar order (fig. S8). To eliminate the incorporation of WT-Mat entirely, we introduced two premature stop codons into the *mat* gene on the WT-ΦCb5 plasmid. This modification effectively eliminates the production of WT-Mat, thereby preventing competition for cellular resources with the engineered phage particles (fig. S9).

## DISCUSSION

In this study, we uncover the structure of the *C. crescentus* Tad pilus, shedding light on its assembly mechanism. Beyond the hydrophobic interactions between pilins to stabilize the pilus, we identify a core network of hydrogen bonds. Notably, the residues, Glu^19^ and Tyr^20^, responsible for these hydrogen bonds are conserved across Tad pilins (fig. S1C), implying a universal mechanism for their involvement in pilus assembly. These hydrogen bonds may be evolved as compensatory elements, making up for the absence of additional pilin-pilin interactions seen in the globular β sheet domains of other type IV pilins. A Glu residue is conserved at the same location in other mature type IV pilins, including the bacterial type IVa and archaeal type IV (fig. S1A). This negatively charged Glu in type IVa pilin is proposed to neutralize the positive charge from the amide group at the N terminus of neighboring type IVa pilins (*12*, *27*, *28*), rather than forming hydrogen bonds with the neighboring pilin as in Tad pili. This highlights how the conserved amino acid adapts to different structural mechanisms for stabilizing various types of pili. In addition, such a network of hydrogen bonds formed by pairs of polar residues in adjacent pilin subunits gives rise to the unique helical symmetry of the Tad pilus assembly.

The structures in our study pinpointed regions on ΦCb5 Mat that bind to the Tad pilus and the U1 helix in the 3′ untranslated region of the gRNA. These findings imply the potential use of this U1-Mat connection for delivering foreign RNA into host bacteria through pilus retraction. Furthermore, we have revealed that the N terminus of Mat is exposed on the surface of the virion, presenting an alternative site, other than the Coat, to display peptides or proteins (for instance, a GFP) on the phage without compromising capsid integrity. This offers a unique opportunity to present a single protein of interest on individual phage particles, as opposed to displaying multiple copies via the Coat (178 Coat proteins per mature virion). This unique feature enhances adaptability and precision in phage display, especially in the later stages when proteins are displayed in lower quantities to improve the affinity between the displayed protein and the target. In contrast to the M13-based DNA phage display, which depends on the secretion of filamentous virions bearing displayed proteins through a specific channel in the *E. coli* cell envelope (*29*), thereby limiting the variety of displayable proteins (*30*), ssRNA phage capsid proteins, including Mat, directly assemble around the genomic RNA, eliminating the necessity for secretion to allow more flexible display of proteins. In addition, ssRNA phages can be produced in vitro (*31*), offering the possibility of cell-free display, which removes the limitations related to transformation efficiency in cell-based systems (*32*).

In our current two-plasmid system, only the wild-type genomic RNA is packaged into the phage particles through the specific interaction between Mat and U1 RNA stem-loop. To establish a connection between the genotype and phenotype of the displayed protein for use in a selection assay, one may incorporate the Mat-binding U1 into the plasmid encoding the displayed protein while using the other plasmid as a “helper phage plasmid.” Our study successfully demonstrated the display of GFP on the Mat of ssRNA phages. To further establish the broad applicability of this display strategy, additional proteins of diverse sizes and folds need to be tested.

Currently, there is a scarcity of effective virus-like particle (VLP) vaccine display platforms, primarily attributed to the steric challenges posed when integrating foreign peptides at either the N or C terminus of each modified Coat (*33*–*35*). Our method for displaying proteins on the Mat offers an option for the ssRNA phage VLP system that had previously relied exclusively on Coat proteins display. This proves advantageous in occasions where Coat protein display results in Coat misfolding and VLP instability (*36*). While it is true that displaying proteins or peptides on the single Mat, as opposed to Coat proteins, provides lower valency and potentially lower immunogenicity for vaccine applications, our structural analysis reveals that the Mat binds to the U1 stem-loop and replaces one Coat dimer at the twofold axis of the phage capsid. This suggests the possibility of introducing multiple U1 RNA stem-loops into the phage RNA, enabling the incorporation of multiple copies of Mat into a VLP to enhance the valency of displayed proteins.

The introduction of GFP-ΦCb5 in this study allows for the visualization of virion and pilus colocalization during fluorescent imaging, offering the potential to track, in real time, the entry of Mat as it escorts gRNA into the cell—a process that has yet to be characterized (*37*). However, the brightness of a single GFP might be insufficient for visualizing inside the bacteria. To overcome this challenge, a possible solution is to incorporate a linker with multiple cysteines (*26*) at the N terminus of Mat, enabling the attachment of multiple dye labels to enhance fluorescence intensities. This capability provides invaluable tools for future research, particularly in unraveling the complexities of the ssRNA phage-host entry mechanism by allowing for a more in-depth examination of interactions, including those between Mat and the basal body from which the pilus extends (*38*).

## MATERIALS AND METHODS

### Preparation of ΦCb5 and bNY30a Tad pili

For phage ΦCb5 amplification, 1 liter of bNY30a cells was grown at 30°C in PYE media [peptone (2 g liter^−1^), yeast extract (1 g liter^−1^), 1 mM MgSO_4_, and 3 mM CaCl_2_] until OD_600_ (optical density at 600 nm) = 0.4. Phage ΦCb5 were added at an MOI (multiplicity of infection) of 10 and cultured overnight. The cells were collected by centrifugation at 4000*g* for 30 min and lysed in 50 ml of TMC3 buffer [20 mM tris, 3 mM CaCl_2_, and 2 mM MgCl_2_ (pH 7.5)] supplemented with one tablet of protease inhibitor (Roche). Lysate was cleared by centrifugation at 4000*g* for 30 min and further cleaned by centrifugation at 15,000*g* for 30 min. Supernatant was concentrated by a 100-kDa cutoff concentrator until volume reaches 1 ml. The sample was then placed on sucrose gradient (20 to 60%) for ultracentrifugation overnight at 200,000*g*. Fractions of phages were collected and dialyzed three times against the TMC3 buffer in the 3-kDa cutoff dialysis cassettes. The sample was then concentrated into 500 μl by the 3-kDa cutoff concentrator and lastly separated by gel-filtration chromatography. All these steps are performed at 4°C unless specified. The purified phages were flash frozen in liquid nitrogen and stored at −80°C for later analysis and experiments.

For bNY30a Tad pili collection, 1 liter of bNY30a cells was cultured to stationary phase at 30°C in PYE media. Cells were then centrifuged down at 4000*g* for 30 min and resuspended in 40 ml of TM buffer [20 mM tris and 2 mM MgCl_2_ (pH 7.5)]. The sample was forced to go through a 23G needle three times for pili shearing. The cell debris was centrifuged down at 15,000*g* for 10 min, and supernatant was concentrated to 2 ml. The CsCl-gradient purification was applied to the sample by ultracentrifugation at 200,000*g* for 24 hours. Each fraction of samples was collected followed by multiple times of dialysis against the TM buffer. All these steps are performed at 4°C unless specified. Negative-staining imaging was performed to check the purity of pili.

### Preparation of the GFP-ΦCb5

The full-length ΦCb5 cDNA was synthesized and cloned into a pET28^+^ vector (Genscript). The codon-optimized GFP-Mat was synthesized (Genscript) and cloned into a pBAD33 vector. For the expression and purification of the GFP-ΦCb5 complex, full-length ΦCb5 plasmid and GFP-Mat plasmid were cotransformed into the *E. coli* BL21(DE3) cells. Five milliliters of cells was induced by 0.5 mM isopropyl-β-d-thiogalactopyranoside and 0.2% l-arabinose at OD_600_ = 0.5. After culturing at 16°C for 16 hours, the cells were collected by centrifugation at 4000*g* for 5 min and resuspended in 800 μl of TMC3 buffer. After the cells were lysed, the solution was cleared by centrifugation at 4000*g* for 5 min. The supernatant was further cleaned by passing through a 0.22-μm syringe filter. The sample was then mixed with 100 μl of streptavidin agarose beads (EMD Millipore), which are conjugated with biotin-labeled Avi-small ubiquitin-like modifier (SUMO)-GFP nanobody. After incubating overnight, the beads were pelleted and washed three times with TMC3 buffer to exclude unbound samples. The beads with bound samples were incubated with an adequate amount of SUMO protease for 30 min followed by TMC3 buffer washing to elute GFP-ΦCb5 complex. All these steps were performed at 4°C unless specified. The purified GFP-ΦCb5 was flash frozen in liquid nitrogen and stored at −80°C for later analysis and experiments. To disrupt the *mat* gene on the native ΦCb5 genome plasmid, site-directed mutagenesis was used to introduce two premature stop codons. One replaced the start codon of mat, and the other replaced the Tyr^180^ in the middle of the *mat* gene (540 base pairs downstream of the original start codon) (fig. S9).

### Cryo-EM sample preparation and data acquisition

The bNY30a Tad pilus was prepared by applying 3 μl of purified sample to glow-discharged holey carbon grid (C-flat, R2/1, 300 mesh) and vitrified using a Vitrobot Mark III (FEI Company) after blotting for 3 s at 6°C with 100% relative humidity. Cryo-EM images of the pili were recorded under a Titan Krios G3 microscope (Thermo Fisher Scientific) equipped with a Gatan Quantum energy filter (silt width, 30 eV) at 300 kV. Automated data acquisition was achieved on a K2 Summit direct detection camera (Gatan) in the super-resolution mode with a pixel size of 0.42 Å and defocus ranged from −0.5 to −2.5 μm. The beam intensity was adjusted to a total electron dose of 42 e^−^/Å^2^. A total of 8980 movie stacks were recorded.

The purified bNY30a Tad pilus and purified ΦCb5 particles were reconstituted in two steps. First, the ΦCb5 particles were concentrated to 20 mg ml^−1^ in a volume of 50 μl by a 3-kDa cutoff concentrator. The pili were concentrated by the 3-kDa cutoff concentrator in a volume of 10 μl. After mixing adequate amount of ΦCb5 and pili sample and incubation on ice for 30 min, 3 μl of the assembled ΦCb5-pilus complex was loaded to a glow-discharged holey carbon (C-flat, 2/1) and vitrified using Vitrobot Mark III for 3-s blotting at 6°C with 100% relative humidity. Cryo-EM images of the ΦCb5-pilus complex were recorded under a Titan Krios G3 microscope (Thermo Fisher Scientific) equipped with a Gatan Quantum energy filter (slit width, 20 eV) and operated at 300 kV. Automated data acquisition was achieved on a K3 Summit direct detection camera (Gatan) with a pixel size of 0.86 Å and a defocus range of −0.5 to −2.5 μm. The beam intensity was adjusted to a dose rate of roughly 1.25 e^−^/Å^2^ per frame for a 40-frame movie stack with a total exposure time of 2.2 s. A total of 39,171 video stacks were recorded for the complex.

### Cryo-EM data processing and structure determination

For the Tad pilus structure determination, super-resolution video stacks of pilus were motion-corrected and binned 2× by Fourier cropping using MotionCor2 (*39*). The micrographs were primarily processed following the workflow cryoSPARC v.3.0 (*40*). The data processing diagram is shown in fig. S2. Contrast transfer function determination was performed using patchCTF. Iterative filament tracer following two-dimensional (2D) classification was used to produce a high-quality dataset for 351,794 particles of pixel size 0.86 Å. The same workflow was performed with the ΦCb5-pilus complex, producing 40,700 filament particles. After merging these two datasets, these particles were subjected to either iterative helical refinement or ab initio reconstruction until visible structural features were observed. A pair of self-calculated helical rise and twist parameters were then input into symmetry search to efficiently narrow the searching range using cryoSPRAC v3.0. The predicted rise and twist parameters were subjected to helical refinement until a decent map was generated. Iterative helical refinement followed with CTF refinement was performed, producing a map with an indicated global resolution of 2.8 Å at a Fourier shell correlation of 0.143.

The data processing scheme for the ΦCb5 virion and the ΦCb5-pilus complex is shown in fig. S4. A total of 39,171 movie stacks were motion-corrected using MotionCor2 and processed following the workflow in cryoSPARC v.4 and RELION3.0 (*41*). Contrast transfer function determination was done by patchCTF. Automated particle selection yielded two datasets of 14,390,103 particles in total, which were extracted on a binned dataset with a pixel size of 3.44 Å. 2D classification was used to remove only “junk” particles (e.g., ice and noncentered particles), producing two datasets of 443,384 and 903,758 good particles, respectively. To further determine a Mat structure, the two datasets were merged into 1,347,142 particles. Ab initio reconstruction and heterogeneous refinement are done with these particles for three classes, producing two high-quality subsets of 1,240,926 particles. These particles were reextracted with a pixel size of 1.72 Å and subsequently subjected to RELION3.0 auto refinement. Further cleaning of the dataset was accomplished by iteratively focused 3D classification with the “skip-align” option. The best subset showing clear Mat structural features was extracted with a pixel size of 0.86 Å. These particles were subjected to RELION3.0 for focused 3D auto refine generating a focused cryo-EM map of Mat and feedbacked to cryoSPRAC v.4 nonuniform refinement obtaining an overall ΦCb5 cryo-EM map. DeepEMhancer (*42*) was used to further sharpen these two maps. For ΦCb5-pilus complex structure determination, a total of 107,432 particles were manually picked by cryoSPARC 4.0 manual pick. One round of heterogeneous refinement was done with these particles for three classes. The best subset of 63,943 particles was reextracted with a pixel size of 0.86 Å and subjected to RELION3.0 auto refine. A 3D classification with skip-align option was performed, producing one best subset of the total 56,330 particles. Iterative focused 3D auto refine was done with these particles, and a final map was generated with DeepEMhancer sharpening. The file conversion between cryoSPARC and RELION3.0 was achieved using UCSF pyem (*43*). The statistics for data collection and processing are listed in table S1.

### Atomic model building and refinement

To build the model of all structures in this study, initial models of the Mat and single pilin were predicted by Alphafold2 (*44*) and manually docked into our cryo-EM map. The capsid shell was fitted with the previous ΦCb5 Coat structure (Protein Data Bank: 2W4Z) using UCSF ChimeraX (*45*). Iterative manual adjustment and rebuilding of models were done with ISOLDE (*46*) and Coot (*47*), which was followed by the real-space refinement in PHENIX (*48*).

For the model of the Mat-Tad complex, the high-resolution structures of the Tad pilus and Mat were first docked into our map of the Mat-pilus complex at 6.4-Å resolution (fig. S4C). The clear secondary structural features allow us to confirm the correct positions of these two models. The molecular interface was then interactive adjusted using ISOLDE and Coot followed by the real-space refinement in PHENIX. All structural figures were prepared using UCSF ChimeraX. The statistics for the models are listed in table S1.

### Testing NA1000 for susceptibility to ΦCb5

To assess NA1000’s susceptibility to ΦCb5 infection, we conducted both standard double agar overlay and spot assays in PYE. Given that NA1000 failed to produce plaques in these assays, we proceeded to examine its phage production capabilities in a liquid culture following the ΦCb5 sample preparation protocols.

Phage presence was monitored in growing lysates at multiple time points 2, 4, 8, and 24 hours using the standard double agar overlay assay on bNY30a, a host known to form plaques. While bNY30a showed an increase in plaque-forming units (PFU) over time, NA1000 did not.

### Phage adsorption assay

ΦCb5 adsorption was assessed using a standard bacteriophage adsorption assay (*49*) with slight modification for adaptation to ssRNA phages. Briefly, starter cultures were initiated from single colonies and grown to saturation in PYE. Experimental cultures were prepared by diluting the starter cultures 1:1000 into fresh medium. These cultures were grown until they reached the exponential phase, and cells were then normalized to 2.0 × 10^8^ CFU ml^−1^. For the adsorption assay, 300 μl of the normalized cell culture was mixed with 100 μl of ΦCb5 lysate at a concentration of 2.0 × 10^7^ PFU ml^−1^, resulting in an MOI of approximately 0.03 providing ample opportunity for ΦCb5 binding. A no-cell control was included in each assay by mixing 300 μl of PYE medium with the phage lysate. Adsorption was conducted in a microcentrifuge tube placed in a 30°C water bath for 10 min. Subsequently, the cells were pelleted by centrifugation at 4000*g* for 2 min to separate bound ΦCb5, and 100 μl of the supernatant collected representing unbound ΦCb5 was transferred to a fresh microcentrifuge tube where PFUs were assessed using a standard double agar overlay plaque assay with the permissive host bNY30a (*15*). Plates were incubated at 30°C to allow for plaque formation. All experiments were conducted in technical triplicate, and the data presented are from at least three biological replicates for each genotype, alongside an internal no-cell control.

### bNY30a pilin sequencing

To compare the pilA gene sequences in bNY30a and NA1000 strains, we designed custom primers (MilliporeSigma) for Sanger sequencing (Eton Biosciences) to span the pilA locus using the published genomes of NA1000 and the CB13B1a, which is the parent to bNY30a. The obtained sequences were aligned using MAFFT version 7 for analysis. Our bNY30a pilA sequence showed >98% sequence identity across multiple runs compared to its reference sequence CB13B1a, with no mutations leading to amino acid substitutions. The NA1000 sequence matched its reference sequence identically.

### Fluorescent imaging of GFP-ΦCb5

To assess the binding of the GFP-ΦCb5 construct to cells, cell samples were grown to exponential phase and normalized to an OD_600_ = 0.1. Cells were then incubated with GFP-ΦCb5 at 30°C for 10 min. The sample (1.2 μl) was placed onto a 1.5% PYE agarose pad and dried for 1 min. The agarose pad was then placed onto a #1 (Fisher Biosciences) 24 mm by 50 mm coverslip for imaging.

For samples that underwent pili labeling, strains were grown and normalized as above before labeling and infecting. Cells were labeled by adding Alexa Fluor-594 C5-maleimide (Thermo Fisher Scientific) to a final concentration of 25 μg ml^−1^ and incubated in the dark for 10 min at 30°C. Cells were washed twice with PYE by centrifugation at 4000*g* for 2 min using a cut tip to reduce shearing stress on pili. Last, cells were resuspended in PYE media and split into two tubes where they were infected with ΦCb5 or an equivalent volume of phage buffer before imaging.

Imaging was performed on a Nikon Eclipse Ti2 inverted epifluorescence microscope using a 100× objective (Plan Apochromat, numerical aperture 1.45, oil immersion) at room temperature and acquired on either a cooled electron-multiplying charge-coupled device camera with mask (Princeton Instruments) or a complementary metal-oxide semiconductor camera (ORCA-Fusion, Hamamatsu Photonics). Cells were imaged under phase-contrast and with the following fluorescent filter cubes: ET GFP (Chroma 96362) and Red (Chroma 49310). A series of five to nine Z stacks of 200 nm were used to allow for Nyquist sampling and capture images of cell-associated pili. Samples were imaged on multiple stage positions (*n* > 10). Camera exposures were as follows: 100 ms for phase contrast to detect cells, 300 ms in the green channel to detect GFP-ΦCb5, and 50 ms in the far-red channel to detect Alexa Fluor 594–labeled pili. Images were analyzed using NIS elements software. When presenting microscopy images in figures, uniform contrast settings were applied for each separate channel throughout the entire figure.

### Statistical analysis

To ensure the robustness of our findings, we used statistical analyses described below. Before conducting *t* tests, we first assessed the normality of both the original and normalized data using the Shapiro-Wilk test and verified the assumption of equal variances using Levene’s test. Both tests confirmed that the data met the necessary assumptions for proceeding with *t* tests.
