# Supplementary material for: Structural mechanisms of Tad pilus assembly and its interaction with an RNA virus
Source: Sci Adv. 2024 May 3;10(18):eadl4450. doi: 10.1126/sciadv.adl4450 (PMC11067988; doi:10.1126/sciadv.adl4450)
Supplement: Supplementary file 1 — Figs. S1 to S9 Table S1 [file sciadv.adl4450_sm.pdf]

Science Advances
AAAS

Supplementary Materials for

Structural mechanisms of Tad pilus assembly and its interaction with an RNA virus

Yuhang Wang *et al.*

Corresponding author: Junjie Zhang, junjiez@tamu.edu

Sci. Adv. **10**, eadl4450 (2024)
DOI: 10.1126/sciadv.adl4450

This PDF file includes:

Figs. S1 to S9
Table S1

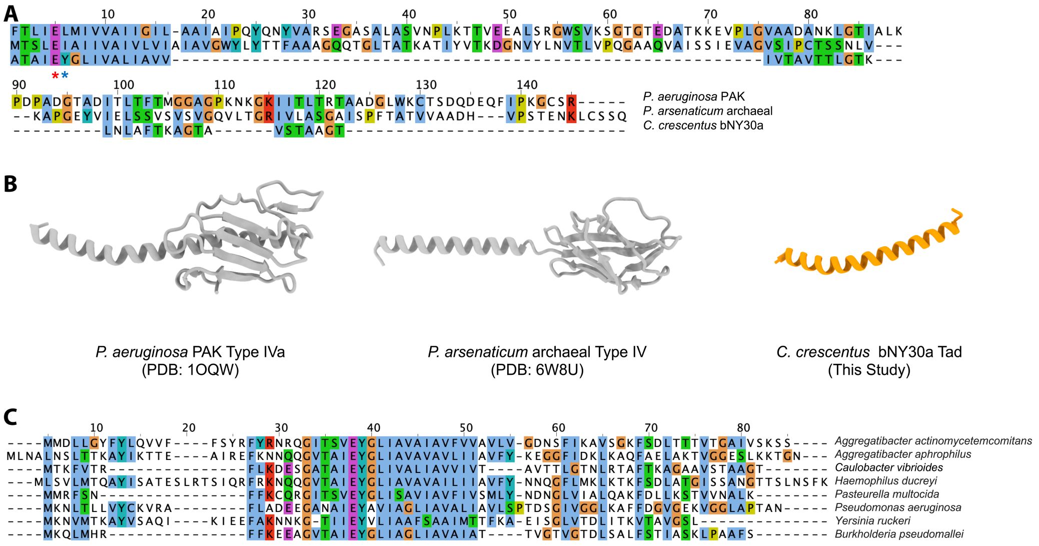

Fig. S1. Sequence and Structural Comparisons among Pilins of Bacterial Type IVa, Archaeal Type IV, and Tad. (**A**) Sequence alignment of the mature Type IVa pilin from *P. aeruginosa* PAK, the archaeal Type IV pilin from *P. arsenaticum*, and the mature Tad pilin from *C. crescentus* bNY30a. While the Glu is conserved (labeled by a red star), the Tyr is not (labeled by a blue star). (**B**) Structural comparison of these three mature pilins in (A). These pilins exhibit a similar N-terminal helical region. (**C**) Alignment of Tad pilin sequences from different bacterial species. The Glu19 and Tyr20 involved in hydrogen-bonding are conserved and labeled by red stars. The prepilin cleavage site at Gly is labeled by black star.

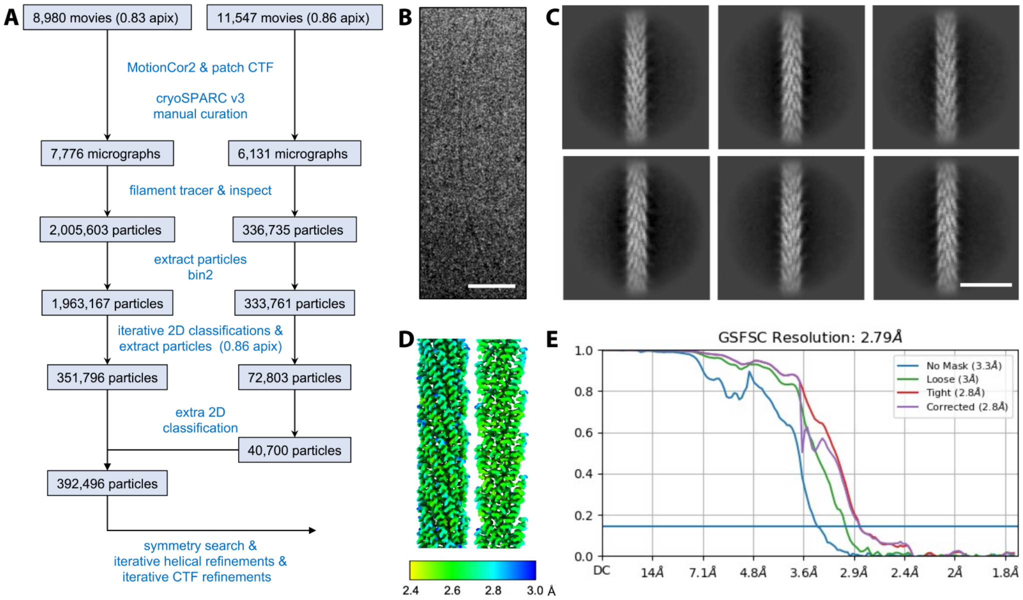

Fig. S2. Cryo-EM data processing workflow of the Tad pilus. (**A**) Cryo-EM data processing diagram of the Tad pilus. (**B**) A representative view of the micrograph after motion correction. The white scale bar denotes 500 Å. (**C**) Selected 2D class averages of the Tad pilus. The white scale bar denotes 100 Å. (**D**) Local resolution estimations of the cryo-EM density map of the Tad pilus showing both surface (left) and cut-open (right) views. (**E**) The gold-standard Fourier Shell Correlation (FSC) curves of the density map of the Tad pilus.

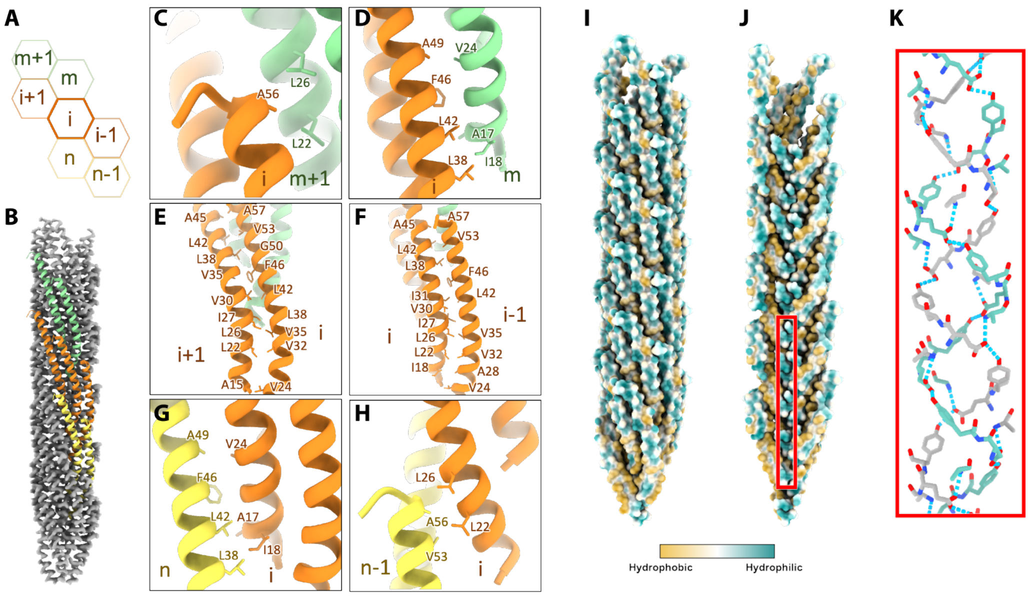

Fig. S3. Hydrophobic interaction and hydrogen bonds within the Tad pilus. (**A**) A schematic diagram showing one pilin and six neighboring pilins. (**B**) The same interacting pilins, denoted in panel A, are colored on the pilus model. (**C-H**) Close-up views, showing the residues responsible for the hydrophobic interaction between neighboring pilin pairs. (**I-J**) Hydrophobicity analysis of the Tad pilus in the surface view (I) and cut-open view (J). (**K**) A close-up view of the red box in (J) showing hydrogen bonds (dashed blue lines) network of Glu19-Ala17 and Glu19-Tyr20 in the core of pilus.

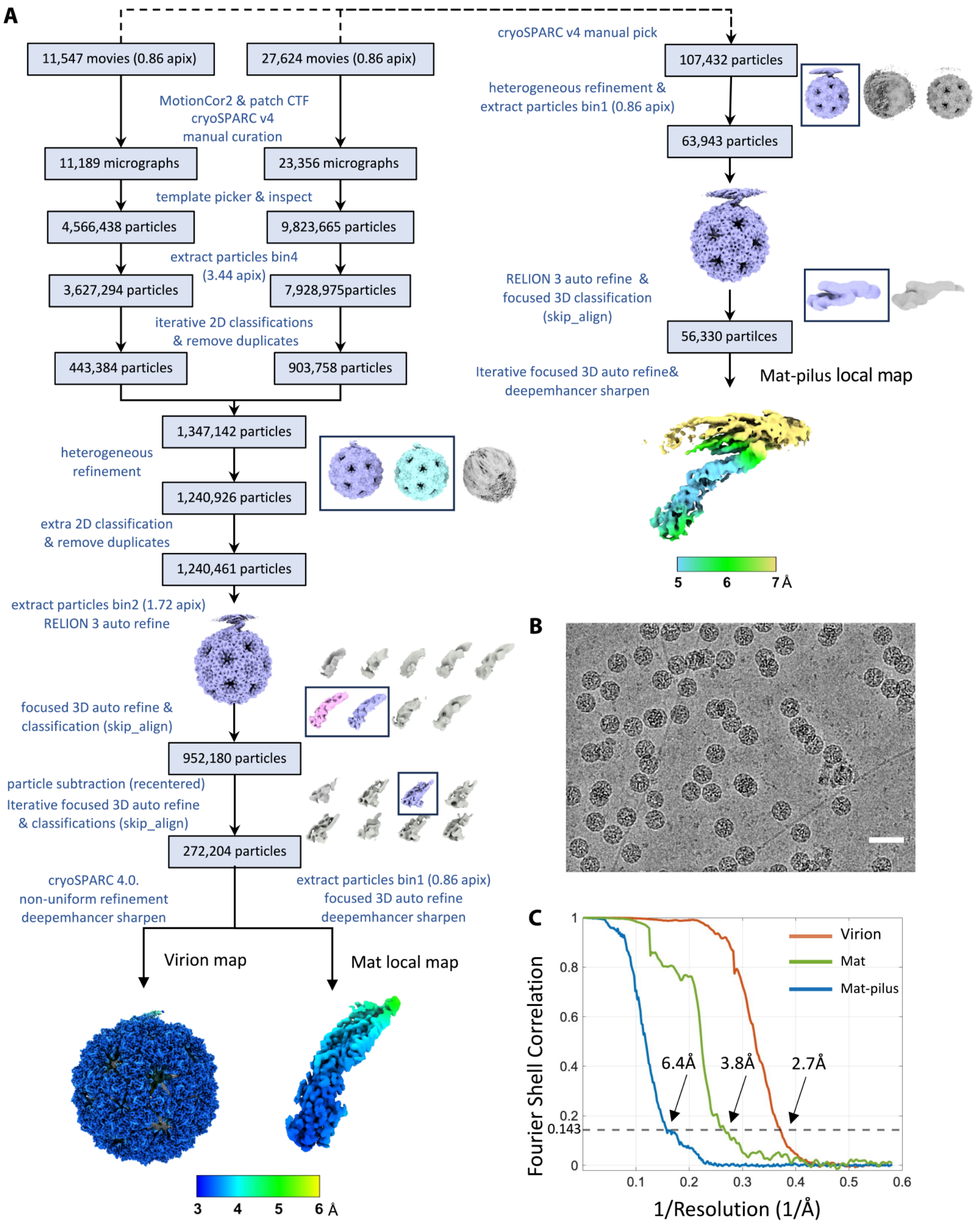

Fig. S4. Cryo-EM data processing workflow of ΦCb5 virion and ΦCb5-Tad complex. (**A**) A diagram showing the cryo-EM data processing workflow. Local resolution of the Virion, Mat, and Mat-pilus maps are color coded. (**B**) A representative cryo-EM micrograph ΦCb5 mixed with Tad pili. The white scale bar denotes 500 Å. (**C**) Gold-standard Fourier Shell Correlation plot showing overall resolutions for the Virion map, Mat local map and Mat-pilus local map, respectively.

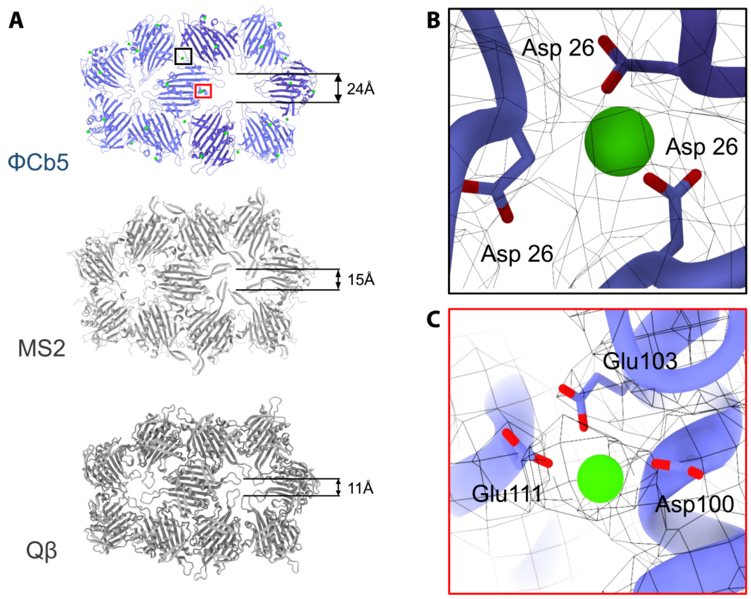

Fig. S5. Unique characteristics of the ΦCb5 Coat shell and the calcium ions in the ΦCb5 capsid. (**A**) The ribbon model depicts adjacent pentamer and hexamer of coat proteins of ΦCb5 (top row), MS2 (middle row) and Qβ (bottom row), respectively. The diameter of the opening at the center of hexamer is labeled. (**B-C**) Zoom-in views of the density and model in the black (B) and red (C) boxed regions on the ΦCb5 Coat shell depicted in (A). Negatively charged amino acid sidechains interact with calcium ions.

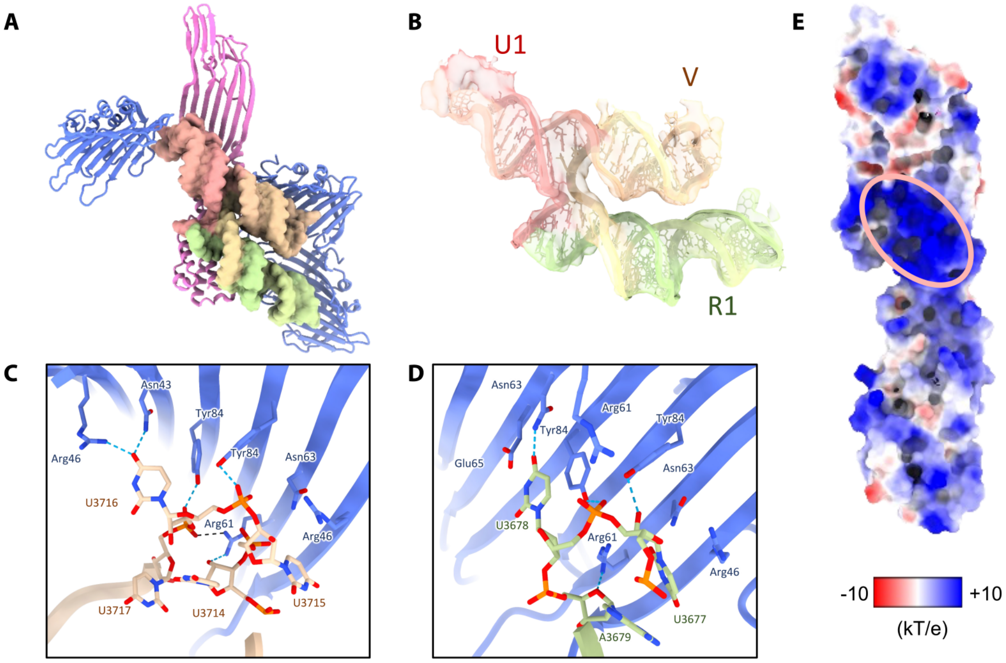

Fig. S6. Interaction of the capsid proteins with the RNA four-way junction domain at the 3' end of the gRNA. (**A**) The overall interaction 3' end domain (colored density) interacts with Mat (pink model) and Coat dimers (blue models). (**B**) Cryo-EM density of the RNA domain matches a four-way junction model of RNA residues 3652-3762. The three long RNA stem-loops are labeled R1, V and U1, respectively. (**C-D**) Interaction between the Coat dimer with Stem-loops V (C) and R1 (D), respectively. Hydrogen bonds and salt bridge are labeled with blue and black dashed lines, respectively. (**E**) Electrostatic surface potential of the Mat showing the U1 binding region (salmon circle) is highly positively charged.

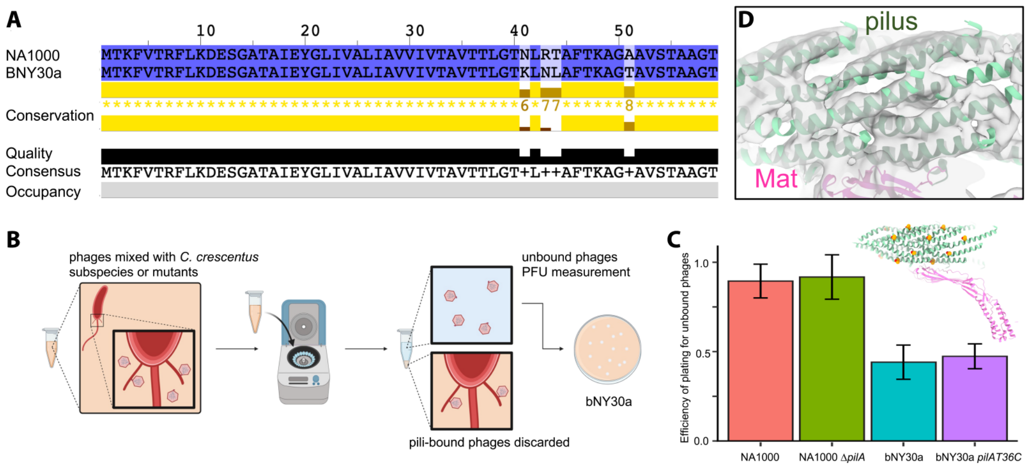

Fig. S7. Adsorption of ΦCb5 with *C. crescentus* subspecies and mutants. (**A**) Sequence comparison of pilins between of NA1000 and bNY30a strains. (**B**) A scheme showing the procedure of the adsorption assay. Briefly, ΦCb5 are incubated with *C. crescentus* subspecies and mutants, allowing pilus binding to occur. The sample is then centrifuged to collect the unbound ΦCb5 phages in the supernatant to further infect the host strain bNY30a. The efficiency of plating (EOP) of the unbound ΦCb5 to bNY30a then be measured. Schematic illustration was created with BioRender. (**C**) EOP of the unbound ΦCb5 showing ΦCb5 binds poorly to NA1000 strain as to the Tad major pilin knock-out strain, NA1000 *ΔpilA*. However, ΦCb5 binds to a pilin-modified strain, bNY30a *pilAT36C*, as good as the host strain bNY30a. In the top right inset, the sites for the cysteine mutation for the amino acid T36 of the pilins are labeled as gold spheres on the pilus model and reside outside the Mat-pilus interface. The T36C mutation allows maleimide dye labeling for the fluorescence imaging. (**D**) Model fitting in map to show the match of pilin models in our cryo-EM density.

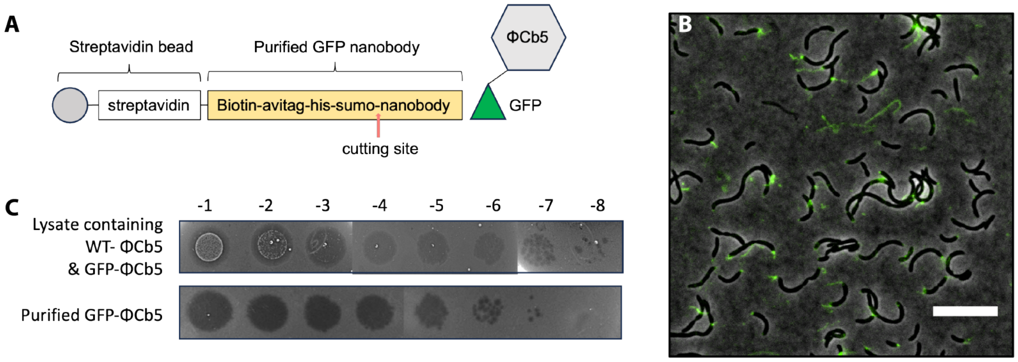

Fig. S8. Purification, fluorescent labeling, and infectivity test of GFP-ΦCb5. (**A**) The anti-GFP nanobody used in the affinity purification of the GFP-ΦCb5. (**B**) Fluorescence imaging of GFP-ΦCb5 binding to bNY30a cells. The white scale bar denotes 10 µm. (**C**) The titer result of the lysate containing the mixture of WT-ΦCb5 and GFP-ΦCb5 was compared with that of purified GFP-ΦCb5. Numbers indicate the dilution of the phage sample by a multiple of ten from left to right. Our findings indicate that the purified GFP-ΦCb5 (titer of ~5×10^9 pfu ml^{-1}) has a one-log drop in titer compared to the mixture (titer of ~8×10^{10} pfu ml^{-1}). Recognizing that the purification process may result in some loss of GFP-ΦCb5, we estimate that the original particle numbers of WT-ΦCb5 and GFP-ΦCb5 are on the same order in our two-plasmid system. In addition, the plasmid for GFP-Mat (pBAD33) is of a lower copy number compared to the plasmid of WT-ΦCb5 (pET28+) in our two-plasmid system. Given the comparable amounts of GFP-ΦCb5 with WT-ΦCb5, we estimate the incorporation efficiency of the GFP-Mat into infectious phage particles should be at least not worse than the WT-Mat.

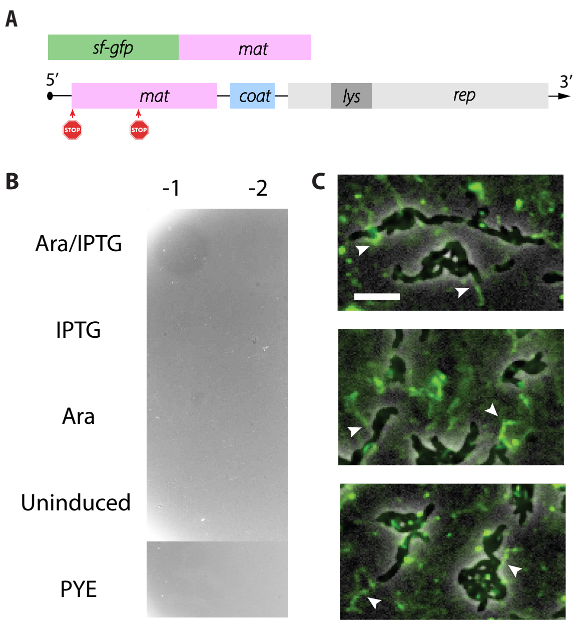

Fig. S9. The modified two-plasmid system shows infectivity and fluorescent binding of GFP-ΦCb5 to Tad pili of *C. crescentus*. (**A**) The modified two-plasmid system introduces two premature stop codons to inhibit the *mat* gene on the WT-ΦCb5 genome. The stop signs annotate the locations of the two premature stop codons: one replaces the start codon while the other is inserted 540 bp downstream the start codon in the middle of the *mat* gene. (**B**) As this new version of GFP-ΦCb5 contains phage RNA with the *mat* gene knocked out, it can still bind the host, deliver the RNA and lyse the cell, but not able to produce new infectious virions inside the cell for subsequent infection of new cells as in a standard titer assay. Therefore, we have performed the following assay with proper controls. The *E. coli* cells with our two-plasmids, encoding the GFP-Mat (pBAD33) and Mat-knockout ΦCb5 genome (pET28+), were induced differently (from top to bottom in Panel B): by both Arabinose (Ara) and IPTG, by IPTG only, by Ara only, uninduced. These *E. coli* cells were then washed and lysed within PYE media, with the lysate applied to *C. crescentus* plate lawns. We observed only the lysate with both plasmids induced, containing these modified GFP-ΦCb5 particles, shows clearing of the *C. crescentus* lawn. Numbers indicate the log dilutions of the sample by a multiple of ten from left to right. Bottom row is another control by applying the PYE media alone onto the *C. crescentus* lawn, which showed no *C. crescentus* killing. (**C**) Fluorescence imaging of modified GFP-ΦCb5 binding to *C. crescentus* bNY30a cells. White arrowheads point to representative Tad pili from the cells. The white scale bar denotes 5 μm.

	pilus (EMDB-41844) (PDB 8U2B)	Mat-Pilus (EMDB-42136) (PDB 8UCR)	ΦCb5 (EMDB-42163) (PDB 8UEJ)
**Data collection and processing**			
Voltage (kV)	300	300	300
Electron exposure (e^{-}/Å^2)	~42	~50	~50
Defocus range (μm)	-0.5 to -2.5	-0.5 to -2.5	-0.5 to -2.5
Pixel size (Å)	0.42	0.86	0.86
Symmetry imposed	C1	C1	C1
Initial particle images (no.)	2,005,603	107,432	3,627,294
Final particle images (no.)	351,796	56,330	272,204
Map resolution (Å)	2.8	3.5 overall (6.4 local refine)	2.7 overall (3.8 local refine)
Threshold		0.143	
**Refinement**			
Initial model used (PDB code)	AlphaFold Model	AlphaFold Model	AlphaFold Model & PDB: 2W4Z
Model resolution (Å)			
Map sharpening method	DeepEMhancer	DeepEMhancer	DeepEMhancer
Model composition			
Non-hydrogen atoms	11,396	7,794	172,913
Protein residues	1,665	1,092	22,088
Ligands	0	0	236
B factors (Å^2)			
Protein	57.58	423.03	62.75
Nucleotide	NA	NA	NA
Ligand	NA	NA	60.64
R.m.s. deviations			
Bond lengths (Å)	0.004	0.003	0.004
Bond angles (°)	0.557	0.636	0.704
Validation			
MolProbity score	1.33	1.58	1.82
Clashscore	3.97	11.57	8.80
Poor rotamers (%)	0.00	0.00	0.00
Ramachandran plot			
Favored (%)	100.00	99.05	95.02
Allowed (%)	0.00	0.95	4.97
Disallowed (%)	0.00	0.00	0.00

Table S1. Cryo-EM data collection, refinement, and validation statistics.
